# Human-derived fecal virome transplantation (FVT) reshapes the murine gut microbiota and virome, enhancing glucose regulation

**DOI:** 10.1371/journal.pone.0337760

**Published:** 2025-12-05

**Authors:** Melany Cervantes-Echeverría, Marco Antonio Jimenez-Rico, Rubiceli Manzo, Abigail Hernández-Reyna, Fernanda Cornejo-Granados, Shirley Bikel, Víctor González, Juan Manuel Hurtado Ramírez, Filiberto Sánchez-López, Jonathan Salazar-León, Gustavo Pedraza-Alva, Leonor Perez-Martinez, Adrian Ochoa-Leyva

**Affiliations:** 1 Departamento de Microbiología Molecular, Instituto de Biotecnología, Universidad Nacional Autónoma de México (UNAM), Avenida Universidad, Cuernavaca, Morelos, México; 2 Laboratorio de Neuroinmunobiología, Departamento de Medicina Molecular y Bioprocesos, Instituto de Biotecnología, Universidad Nacional Autónoma de México (UNAM), Avenida Universidad, Cuernavaca, Morelos, Mexico; 3 Programa de Genómica Evolutiva, Centro de Ciencias Genómicas, UNAM, Av. Universidad, Cuernavaca, Mexico; UNAM FACMED: Universidad Nacional Autonoma de Mexico Facultad de Medicina, MEXICO

## Abstract

The gut microbiome, comprising bacteria, viruses, archaea, fungi, and protists, plays a crucial role in regulating host metabolism and health. This study explored the effects of fecal virome transplantation (FVT) from healthy human donors on metabolic syndrome (MetS) in a diet-induced obesity (DIO) mouse model, without diet change. Mice received a single oral dose of human-derived virus-like particles (VLPs) and continued on a high-fat diet (HFD) for 17 weeks. Despite persistent dietary stress, FVT significantly improved glucose tolerance. Longitudinal profiling by virome shotgun metagenomics and bacterial 16S rRNA sequencing revealed marked, durable shifts in both viral and bacterial community composition. Notable bacterial changes included a decrease in *Akkermansia muciniphila* and *Peptococcaceae* and increases in *Allobaculum* and *Coprococcus*; *A. muciniphila* positively correlated with glucose levels and negatively correlated with body weight. Together, these results suggests that human-derived virome can durably reshape gut microbial ecology and improve glucose metabolism in mice with obesity, even without dietary modification, offering a novel avenue for developing phage-based therapies. This proof-of-concept study provides foundational observations for using human-derived VLPs for FVT in standard laboratory mouse models, and provides a foundation for elucidating bacteria-phage interactions and their role in host metabolic health.

## Introduction

Obesity is a growing global health challenge [1,2] that substantially increases the risk of developing metabolic syndrome (MetS) and other chronic conditions [3,4]. While the gut microbiome—particularly its bacterial component—has been extensively studied for its role in host metabolism and obesity-related traits [5], emerging evidence highlights the importance of the gut virome, particularly bacteriophages (phages), in shaping microbial dynamics and influencing metabolic outcomes [6,7]. Phages change bacterial populations through either lytic or lysogenic cycles; in the lytic cycle, they destroy their hosts, while in the lysogenic cycle, they integrate into the bacterial genome as prophages [8]. Changes in the diversity and richness of the gut virome have been associated with obesity and MetS across different age groups [6,7]. Moreover, environmental factors such as high-fat diet (HFD) influence the gut virome structure in humans and animal models [9,10].

Fecal virome transplantation (FVT) is an emerging approach that involves the transfer of virus-like particles (VLPs) from a donor’s fecal sample to a recipient, without the transfer of bacteria. In murine models, FVT from healthy donors has been shown to increase bacterial richness and diversity [11,12], reduce weight gain and improve blood glucose tolerance [11], and even induce lean or obese phenotypes [13]. In humans, FVT has demonstrated potential in improving glycemic control in individuals with MetS [14] and in resolving *Clostridium difficile* infections [15]. However, most FVT studies have focused on within-species transplantation—either mouse-to-mouse or human-to-human.

A recent study demonstrated that human-to-mouse FVT can alter the murine gut microbiota and mitigate inflammatory bowel disease (IBD) symptoms. However, this work utilized a gnotobiotic model colonized with a humanized microbiota [16]. This highlights a gap in current research: the need for studies using conventional murine models to evaluate the impact of human-derived FVT. Such models offer a valuable platform for elucidating bacteriophage–bacteria interactions and understanding how virome manipulation can influence host metabolic health. Here, we investigated the effects of FVT using VLPs isolated from healthy, normal-weight human donors on gut microbial and virome composition and metabolic outcomes in a diet-induced obesity (DIO) mouse model. To our knowledge, this is the first study to evaluate the impact of human-derived FVT in a murine model with a non-humanized gut microbiota. This proof-of-concept study aimed to assess whether FVT could beneficially alter the gut virome and bacteriome while improving metabolic traits associated with obesity and MetS.

## Materials and methods

### Collection of VLPs used in fecal virome transplant (FVT)

Fecal samples were obtained from a previously described cohort of 11 healthy, normal-weight children aged 7–10 years [6]. These fecal samples were drawn from a well-characterized cohort previously established in our laboratory, with standardized, deep sequencing of the microbiota, virome, and metatranscriptome. Each sample includes comprehensive metadata and underwent rigorous quality control, as detailed in our prior publications [5,6,17]. Virus-like particles (VLPs) were isolated using a protocol established by our group [18]. Briefly, ~ 250 mg of each fecal sample was suspended in sterile SM buffer (50 mM Tris-HCl, 8 mM MgSO₄, 100 mM NaCl, pH 7.5), then centrifuged at 4,700 × g at room temperature. The supernatant containing VLPs was filtered through a 0.45 μm PES syringe filter to remove bacterial cells and concentrated to 200 μL using Amicon Ultra-15 centrifugal filter units (100 kDa MWCO, Millipore, UFC910024). Subsequently, 40 μL of chloroform was added and incubated for 10 min at room temperature to disrupt residual bacterial membranes and facilitate the removal of cellular debris. To eliminate free DNA not protected by capsids, samples were treated with DNase I (2.5 U/mL; Invitrogen, Cat. 18047−019). This DNase I was sufficient to remove contaminating host and bacterial DNA prior to DNA-virome sequencing. This step depletes residual extracapsular (non-encapsidated) DNA so that the recovered nucleic acids derive predominantly from within intact viral capsids. Because our library preparation selectively targets DNA, adding RNase offered no substantive benefit, and any residual RNA did not interfere with the workflow. The VLPs were washed five times and re-concentrated in SM buffer using the Amicon filter to retain viral particles and other components larger than 100 kDa. This process was made to enrich intact VLPs while depleting most metabolites and peptides <100 kDa; however, some higher–molecular-weight and protein-bound compounds may persist. VLP purity and quantification were assessed by epifluorescence microscopy using SYBR Green I staining. Microscopy visualization and quantification of VLPs were conducted using an Olympus Multi-photon confocal microscope (plate number: FV1000), following the protocol previously published in detail by our lab [18]. Briefly, we prepared three slides for each sample and captured three non-overlapping micrographs per slide. This resulted in a total of 12 non-overlapping micrographs per sample. We then utilized FIJI software (Schindelin et al., 2012) for image analysis. The protocol used images with dimensions of 0.212 × 0.212 millimeters. We counted the number of fluorescent VLPs in each of the 12 random fields and calculated the average. The absolute number of VLPs was determined based on the average counts per field, factoring in the area of one field and back-calculating with the dilution factor. All fields of the images were validated through manual counting. The image-analysis workflow (FIJI v2.14) included the following steps: images were converted to 8-bit, followed by thresholding. For the “Analyze Particles” function, we specified a size range of 0.02–0.50 µm² and a circularity range of 0.60–1.00. The complete workflow was performed in triplicate per micrograph, and the results were averaged. Counts were converted to particles per gram of feces after correcting for the dilution factor and filter area. Finally, VLPs from all 11 donors were pooled and used as the FVT inoculum.

### Animal study design

All animal procedures followed the protocol previously described by Manzo et al. (2024) to develop the DIO mouse model [19]. Briefly, twelve male C57BL/6N mice (3–4 weeks old) were randomized and fed a standard diet (2018S Envigo Teklad Global Diet: 18% fat, 58% carbohydrate, 24% protein kcal) for four weeks to normalize the gut microbiota. Mice were then assigned to two experimental groups: Control (n = 6) and FVT (n = 6), each housed in two cages of three mice. The individual animal was considered the experimental unit. Both groups were switched to a high-fat diet (HFD; Research Diets D12492: 60% fat, 20% carbohydrate, 20% protein kcal) for 14 weeks to induce obesity and MetS.

All procedures were approved by the Institutional Bioethical Committee, protocol number 300, and conducted according to national and international guidelines for the care and use of laboratory animals. Mice were maintained on a standard diet for eight weeks (four weeks post-birth and four weeks during microbiota normalization), followed by a high-fat diet (HFD) for 32 weeks (14 weeks to establish the DIO model and 18 weeks during the FVT experiment. The selected age range (2–10 months) corresponds to young adult and middle-aged stages, during which mice are not considered senescent. Prolonged HFD feeding for up to 32 weeks was reported to be well tolerated without diet-induced mortality [19–21]. Animal health and welfare were monitored throughout via daily observations, weekly measurements of body weight, food, and water intake, and periodic metabolic evaluations (glucose tolerance and insulin resistance at weeks 0, 10, and 17 post-FVT). No animals met predefined humane endpoints, and all remained within the physiological ranges reported for comparable experimental models.

### Fecal virome transplantation

Mice received an oral gavage of 100 μL of 1.33% NaHCO₃ to neutralize gastric acidity, followed by 200 μL of either saline (Control group) or pooled human-derived VLP suspension (FVT group; 4.4 × 10⁸ VLPs). After transplantation, both groups continued the HFD for an additional 18 weeks. Mice were euthanized at 40 weeks of age according to ethical guidelines.

### Glucose tolerance and insulin resistance tests

Glucose tolerance (GTT) and insulin resistance tests were performed at baseline (immediately before FVT) and at 10 and 17 weeks post-FVT. For GTT, glucose was administered intraperitoneally, and blood glucose levels were measured from the tail vein blood at 0, 15, 30, 60, and 120 minutes. For insulin resistance testing, mice received intraperitoneal insulin (1 U/kg; Humulin R, Eli Lilly), and glucose levels were similarly monitored.

### Fecal sample collection and DNA extraction

For bacterial profiling, fecal samples from four randomly selected mice per group (two mice per cage) were collected at five time points: pre-FVT, and post-FVT on day 1, and weeks 1, 10, and 17. Samples were preserved in RNAlater (Invitrogen) at −70 °C. DNA was extracted using the Quick-DNA Fecal/Soil Microbe kit (Zymo Research). DNA concentration and integrity was evaluated with Qubit fluorimeter (Invitrogen Q33231) and agarose gel electrophoresis. The V3–V4 region of the 16S rRNA gene was amplified using primers 341F and 805R. PCR products (~500 bp) were purified (AMPure XP beads), barcoded, and quantified with a Qubit fluorimeter and Agilent 2100 Bioanalyzer. Sequencing was performed on the Illumina MiSeq platform (2 × 250 paired-end reads) at the National Institute of Genomic Medicine and Unidad Universitaria de Secuenciación Masiva y Bioinformática (IBt-UNAM), Mexico.

### Bioinformatics: Bacterial community analysis

Raw reads were trimmed to remove primer sequences and filtered for quality (Q > 20, length > 350 nt) [22]. High-quality reads (NCBI under BioProject: PRJNA1003883) were joined and processed using DADA2 pipeline (QIIME v2 v2023.9.1) to obtain Amplicon Sequence Variants (ASVs) and compared against the Greengenes database (v13.8). Singletons and ASVs with <0.1% relative abundance were excluded [23]. Alpha diversity was calculated using 10,000 iterations and rarefied at 75% of the smallest sample, and beta diversity was determined using unweighted UniFrac distances. Differentially abundant taxa were identified with DESeq2 in R.

### Viral DNA extraction and sequencing

VLPs were isolated exclusively from fecal samples of the FVT-group collected at baseline (pre-FVT) and at weeks 10 and 17 post-FVT. At each time point, feces were pooled by cage (50 mg per mouse; 2 mice per cage), yielding three pools (n = 3). VLP isolation followed our established protocol [6,18]. Epifluorescence microscopy confirmed the presence of VLPs and the absence of bacterial contamination, using *Escherichia coli* (S1A in S1 Fig) and Rhizobium etli phage φ01 (S1B in S1 Fig) as size references for bacterial and phage, respectively (S1C–F in S1 Fig). Viral DNA from these VLPs was extracted using the QIAamp MinElute Virus Spin Kit (Qiagen, Cat. 57704), quantified with a Qubit (Thermo Fisher, Cat. Q32851), and diluted to 0.3 ng/μL. Libraries were prepared using Nextera XT (Illumina, Cat. FC-131–1024), purified with AMPure XP beads, and sequenced on the Illumina NextSeq500 (2 × 150 pair-end) at the National Institute of Genomic Medicine and Unidad Universitaria de Secuenciación Masiva y Bioinformática (IBt-UNAM), Mexico.

### Bioinformatics: Viral community analysis

Raw reads were trimmed to remove primer sequences and filtered for quality using Trimmomatic [24]. Mice host and bacterial reads were filtered using SMALT (Mus musculus, -c 0.6) and Kraken [25]. Cleaned reads were assembled with IDBA-UD [26] and SPAdes (35–115 k-mer) and merged using METASSEMBLER. Scaffolds <2 kb were discarded. Mapping to contigs was done using SMALT (-c 0.7, -y 0.9). Contigs were retained if they covered ≥70% length at ≥90% identity in at least one sample. CD-HIT (90% identity) was used to remove redundant contigs. We screened the assembly with three independent viral-calling approaches: homology (BLASTx with MEGAN6) [27], genome-quality assessment (CheckV) [28], and machine learning (geNomad) [29]. We retained contigs supported as viral contigs by at least two methods for all downstream analyses. Viral taxonomy at family level was assigned via BLASTx against the NCBI nr viral protein database and analyzed in MEGAN6. Eukaryotic viral contigs were excluded to retain only phage contigs for further analysis. Bray-Curtis dissimilarity was analyzed using the vegan package in R. Alpha diversity (Shannon and Chao1) was calculated with vegan in R (10,000 iterations, rarefied at 75% of the smallest sample), and phage family abundances were normalized by Transcripts per Million (TPM) to account for contig length and sequencing depth and then analyzed with LEfSe. Contig differential abundance was assessed with DESeq2 on raw counts. Spearman correlations between differentially increased contigs post-FVT and the bacterial microbiota were evaluated in R. Phage host prediction was performed with iPHoP v1.4.1 using default parameters and the Jun_2025_pub_rw database [30].

### Statistical analysis

Normality of datasets was assessed using the Shapiro-Wilk test in R. Between-group comparisons for weight, GTT, and insulin resistance were conducted with Student’s t-test. The area under the curve (AUC) for glucose was calculated using the trapezoidal method of the DescTools R package. The beta diversity of bacterial communities was compared with ANOSIM on Unweighted-UNIFRAC distances with 999 permutations, after verifying dispersion with the betadisper function from the vegan R package. Bacterial alpha diversity between groups was compared with Wilcoxon post-hoc tests. Taxonomic differences were identified using DESeq2, applying p-value < 0.05 and log₂ fold change ≥1.5. For virome comparisons, DESeq2 was used with a stricter log₂ fold change ≥2 and adjusted p-value < 0.01 (Benjamini-Hochberg FDR correction). Viral community composition changes over time were analyzed using ANOSIM with 999 permutations on Bray-Curtis distances. Viral Alpha diversity was compared with Wilcoxon post-hoc test and viral families with differential abundance were identified in LEfse, with LDA score >2 and p-value <0.05.

### Human-mouse microbiota comparison

To evaluate overlap in microbial taxa between mice and humans microbiotas, we compared 16S rRNA data from the human VLPs donor cohort [6] against our recipient mice cohort using identical bioinformatic workflows. Venn diagrams were constructed, including taxa with >0.01% relative abundance.

### Ethical approval

The collection of human samples was previously approved and detailed in the article: https://pubmed.ncbi.nlm.nih.gov/32143621/ [5]. Written informed consent was obtained from the parents or legal guardians, along with verbal assent from all participating children. The Institutional Bioethical Committee, protocol number 300 approved all animal experiments described in this study.

## Results

### 1. FVT improves glucose regulation in a DIO mouse model

**Fig 1:**
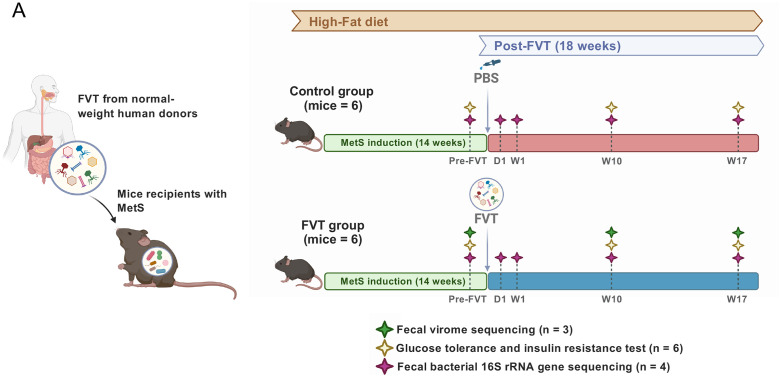
Overview of the Cross-Infection Model Used in This Study. The FVT was conducted by transferring VLPs from normal-weight humans to recipient mice. The experiment involved twelve male C57BL/6 mice, which were fed a high-fat diet (HFD) until they developed DIO mouse model, as was previously reported (18). The mice were then divided into two groups: one group (n = 6) received PBS buffer as a control (Control group), and the other group (n = 6) received the VLPs (FVT group). The HFD was maintained throughout the 18-week experiment, during which the mice’s weights were continuously monitored. Glucose tolerance and insulin resistance were assessed at baseline (pre-FVT) and post-FVT at weeks 10 (W10) and 17 (W17), with n = 6 per time. The fecal bacterial 16S rRNA gene profiles were analyzed at pre-FVT and at day 1 (D1) and weeks 1, 10, and 17 (W1, W10, W17) post-FVT, with n = 4 per time point per groupt. Additionally, the fecal virome was analyzed at pre-FVT and post-FVT at weeks 10 (W10) and 17 (W17), with n = 3 per time, exclusively for the FVT-group. Some image elements were created in BioRender: https://BioRender.com/x17f64q.

Twelve male C57BL/6N mice were fed a high-fat diet (HFD) for 14 weeks to induce obesity and MetS (Fig 1), a DIO mouse model that we previously described [19]. Following this induction period, mice were randomly assigned to two groups for FVT. The FVT group received a single oral gavage of 4.4 × 10⁸ virus-like particles (VLPs), pooled from the fecal samples of eleven healthy, normal-weight human donors previously characterized [6]. In contrast, the Control group received an equivalent volume of phosphate-buffered saline (PBS). Both groups remained on the HFD for an additional 18 weeks post-treatment (Fig 1). Notably, body weight gain and food consumption did not differ significantly between the FVT and Control groups at any time point post-FVT (S2A-S2B in S2 Fig).

**Fig 2:**
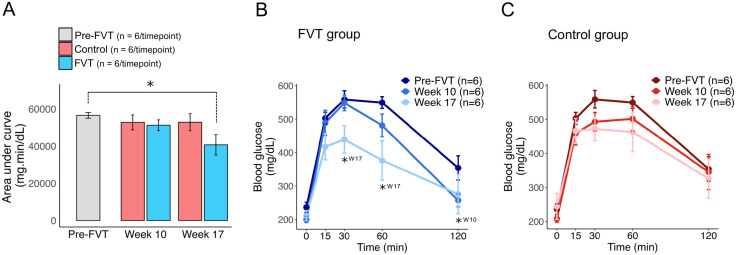
Glucose tolerance test after FVT. (A) Mean area (±SEM) under the glucose curve (AUC, 0-120 min) at baseline (pre-FVT) and at weeks 10 (W10) and 17 (W17) post-FVT in the FVT and Control groups (n = 6 per group, per time point). (B-C) Mean blood glucose (±SEM) during intraperitoneal glucose administration at 0, 15, 30, 60, and 120 min for the FVT (B) and Control (C) groups at baseline (pre-FVT), and at weeks 10 and 17 post-FVT. Statistics: in (A), within-group changes at weeks 10 and 17 versus baseline (pre-FVT) are shown; significance is indicated by * (p < 0.05). Only the FVT group showed significant changes. The between-group (FVT vs. Control) changes at weeks 10 and 17 were not significant. In (B–C), between-group pairwise comparisons (pre-FVT vs W10 and pre-FVT vs W17) across time point (0, 15, 30, 60, and 120 min) are shown at each time point; significance is indicated by * (p < 0.05) and week (W).

To assess metabolic improvements, glucose tolerance tests (GTT) were performed at baseline (pre-FVT), and at weeks 10, and 17 post-FVT for the two groups. Notably, the FVT group exhibited a significant reduction in the area under the curve (AUC) for glucose concentration at week 17 compared to baseline (pre-FVT), indicating improved glucose clearance (Fig 2A). The FVT group also exhibited a significant reduction in the blood glucose levels measured 120 minutes after intraperitoneal glucose administration at week 10 compared to baseline (Fig 2B). The reduction of blood glucose levels was even more pronounced and significant at week 17 (30 and 60 minutes after intraperitoneal glucose administration) compared to baseline and week 10, accompanied by a visibly flattened glucose curve (Fig 2B). These results suggest that FVT conferred a substantial improvement in blood sugar regulation. In contrast, the control group displayed no significant changes in the AUC for glucose concentration (Fig 2A) and in glucose levels at different times after intraperitoneal glucose administration (Fig 2C). Insulin resistance, as assessed by insulin tolerance testing, remained without significant differences in both groups throughout the study time points (S3 Fig).

### 2. FVT induces time-dependent shifts in gut microbiota composition

To evaluate the impact of FVT on gut bacterial communities, we sequenced the V3–V4 hypervariable regions of the 16S rRNA gene from fecal samples collected pre-FVT, and at weeks 1, 10, and 17 post-FVT (Fig 1). After quality control and filtering, an average of 17,720 reads per sample were assigned to 126 ASVs with a relative abundance >0.1% (S1 Table). Richness rarefaction curves demonstrated near-saturation sequencing coverage across all samples (S4 Fig), and Good’s coverage estimation confirmed over 99.85% of taxa were identified. At both the beginning pre-FVT and end week 17 post-FVT of the experiment, bacterial richness and diversity were similar between groups (S5A, S5B in S5 Fig). A transient reduction of richness and diversity was observed in the Control group compared to the FVT group at week 1. However, this decrease was restored by week 10 and was maintained until week 17. When examinig richness and diversity trends (S5 Fig), both the Control and FVT groups showed a similar pattern—a decrease at week 1 followed by a recovery at week 10. This initial drop was more pronounced in the Control group. While this observation was unexpected for the Control group at week 1, a longitudinal comparison across all time points within each group revealed no statistically significant differences in richness or diversity. Therefore, these transient variations did not appear to impact the overall richness and diversity profiles.

**Fig 3:**
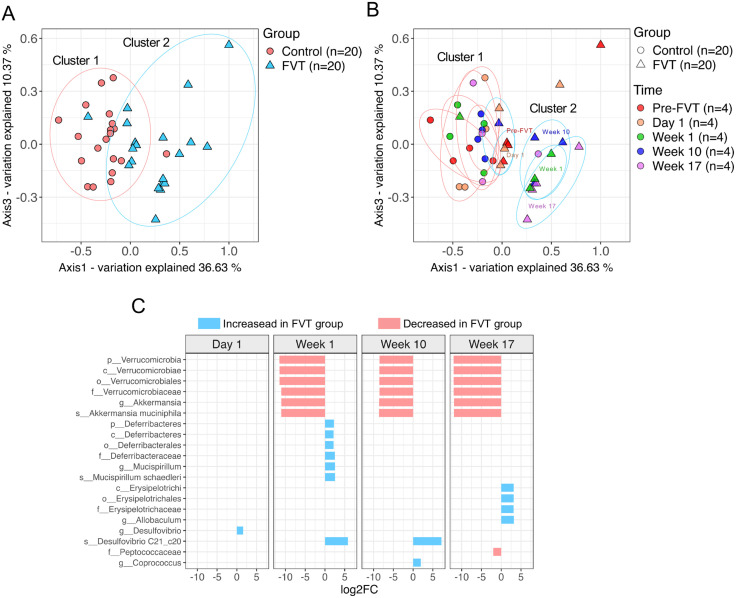
Beta Diversity and Differential Abundance Analysis of Gut Microbiota after FVT. Panels A and B show the Beta diversity analysis of the microbiota based on Unweighted UniFrac distances visualized by Principal Component Analysis (PCoA) plots. (A) Samples grouped by treatment: control (n = 20) vs. FVT (n = 20). (B) Samples categorized by treatment (control Vs FVT) and time point (pre-FVT and post-FVT). For each time point, sample size was n = 4 for the control group and n = 4 for the FVT group. (C) Differential abundance of gut microbiota at the different time points post-FVT. The blue bars indicate taxa significantly increased, while the red bars indicate taxa significantly decreased in the FVT compared to the control. Differentially abundant taxa were identified using DESeq2 with a p-value < 0.05 and a Log2 fold change (Log2FC) cutoff of 1.5. Taxonomic ranks are abbreviated as follows: p for phylum, c for class, o for order, f for family, g for genus, and s for species.

Beta diversity analysis using unweighted UniFrac distances revealed significant differences in bacterial community compositions separating the control (cluster 1) and FVT (cluster 2) samples (Fig 3A). ANOSIM confirmed these significant group differences (R = 0.348, p = 0.001). Notably, most of control samples clustered consistently in cluster 1 across time, indicating minimal compositional drift (Fig 3B). FVT samples at baseline and day 1 also fell within Cluster 1, suggesting no immediate effect of the FVT at day 1 (Fig 3B). ANOSIM confirmed not significant differences between these samples (baseline vs. day 1) in the FVT group (R = 0.135, p = 0.162). In contrast, most FVT samples at weeks 1, 10, and 17 shifted to cluster 2, indicating a pronounced and sustained restructuring of the bacterial community (Fig 3B). ANOSIM confirmed significant differences between the samples at these three time points (weeks 1, 10 and 17) between control and FVT groups (R = 0.435, p = 0.001). These findings suggest that a single FVT dose induced a durable microbiota shift detectable as early as one-week post-treatment and persisting through the 17-week study period (Fig 3B).

Next, we assessed the taxonomic composition and relative abundance of bacterial taxa across all time points for both experimental groups (S6 Fig). Differential abundance analysis revealed 20 taxa that exhibited statistically significant differences between the FVT and Control groups at Day 1, and weeks 1, 10, and 17 post-FVT (Fig 3C). Among these, *Akkermansia muciniphila* exhibited a marked reduction post-FVT and, following week 1 persisted at consistently low abundances. At week 1, its relative abundance was significantly lower post-FVT compared to the Control group. This reduction persisted at weeks 10 and 17(Fig 3C).

In contrast, *Mucispirillum schaedleri* displayed a transient enrichment at week 1 in the FVT group. Desulfovibrio increased abundance at day 1 and Desulfovibrio C21_c20 at week 1 and 10 post-FVT.

Furthermore, Allobaculum was significantly increased at week 17 post-FVT, (Fig 3C). Additionally, Peptococcaceae was significantly decreased at week 17 post FVT (Fig 3C).

### 3. Microbial signatures linked to weight and glucose regulation in FVT-treated mice

**Fig 4:**
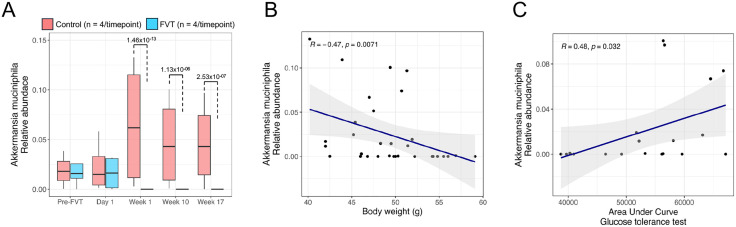
Longitudinal dynamics of *A. muciniphila* and Metabolic Associations. (A) Relative abundance of A. muciniphila at baseline (pre-FVT) and post-FVT (Day 1, Week 1, Week 10, and Week 17) in FVT and control mice (lines = group median ± Interquartile range). (B-C) Spearman correlations between *A. muciniphila* abundance and (B) body weight and (C) the AUC glucose levels in mice.

Among the 20 bacterial taxa identified as differentially abundant between the FVT and control groups (Fig 3C), the dynamics of *A. muciniphila* and Allobaculum were particularly notable. One-week post-FVT, the relative abundance of *A. Muciniphila* had already decreased and was undetectable by week 17 only in the FVT group (Fig 4A). In contrast, its levels steadily increased in the control group over the same period (Fig 4A). This observation suggests a selective suppresion of this taxon associated with FVT. Furthermore, the relative abundance of *A. muciniphila* was negatively correlated with body weight (R = −0.47, p = 0.0071) (Fig 4B) and positively correlated with the AUC glucose levels during the glucose tolerance test (GTT) (R = 0.48, p = 0.032) (Fig 4C). These findings suggest that the depletion of *A. muciniphila* in the FVT group may have had a significant impact on metabolic regulation, particularly on glucose metabolism.

**Fig 5:**
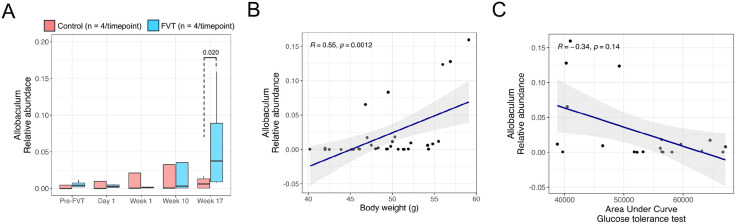
Longitudinal Dynamics of Allobaculum and Metabolic Associations. (A) Relative abundance of Allobaculum at baseline (pre-FVT) and post-FVT (Day 1, Week 1, Week 10, and Week 17) in FVT and control mice (lines = group median ± Interquartile range). (B-C) Spearman correlations between Allobaculum abundance and (B) body weight and (C) the AUC glucose levels in mice.

The relative abundance of Allobaculum gradually increased over time in the FVT group, reaching significance by week 17 post-FVT (Fig 5A). Moreover, Allobaculum abundance correlated positively with body weight (R = 0.55, p = 0.0012; Fig 5B). However, the correlation between Allobaculum abundance and glucose AUC during the glucose tolerance test (GTT) was not significant (Fig 5C).

### 4. Specific virome changes were associated with the FVT

The Viral-like particles (VLPs) were isolated from fecal samples collected from the FVT-treated mice group at three key time points: pre-FVT, and at weeks 10 and 17 post-FVT (Fig 1). Epifluorescence microscopy confirmed the presence of VLPs in the samples (S1 Fig). The total number of recovered VLPs remained consistent across the three experimental time points, obtaining 4.94 × 10^9^, 6.74 × 10^9^, and 9.54 × 10^9^ VLPs per gram of feces for pre-FVT, week 10, and week 17 post-FVT, respectively. This suggests that FVT did not change the number of VLPs in the recipient mice throughout the experiment (S1 Fig).DNA was extracted from the VLPs, and sequence libraries were constructed using a TAG method to mitigate biases often associated with whole genome amplification (WGA), commonly employed due to the low DNA yield from VLPs. After quality filtering, an average of 2,701,536 high-quality paired-end sequences per sample were obtained. To specifically target sequences originating from VLPs, we excluded reads mapped to bacterial (29.63%) and mouse (10.61%) genomes. After filtering, 14,173,277 paired reads were retained, averaging 1,574,808 paired reads per sample, and these were used for virome assembly. Non-redundant meta-assembly resulted in 6,166 contigs greater than 2 kb, which were further filtered to remove 479 eukaryotic virus contigs and 189 contigs with less than 75% coverage in at least one sample, leading to a final virome assembly of 5,498 contigs. Although co-transfer of eukaryotic viruses with the FVT inoculum cannot be fully excluded, two factors make biologically meaningful effects unlikely: (i) the VLPs were human-derived whereas recipients were mice, creating strong host-range barriers, and (ii) the preparation workflow (0.22-µm filtration, chloroform treatment, DNase digestion) is optimized for non-enveloped VLPs and markedly depletes enveloped and large eukaryotic viruses (e.g., Coronaviridae, Herpesviridae). While small, non-enveloped eukaryotic viruses can pass the filters, only 479 of 6,166 contigs (7.8%) were of putative eukaryotic origin, and their abundance was very low (mean 3.1% of quality-filtered reads). Given this low signal and limited statistical power, we restricted downstream analyses to the bacteriophage fraction.

The assembly of the 5,498 contigs had an N50 of 5,776 bp and an L50 of 1,253 bp. On average, 37.75% of the reads per sample were mapped to the assembly. We analyzed the assembled contigs using three complementary approaches to identify viral sequences: (i) protein-level homology searches with BLASTx, followed by taxonomic assignment in MEGAN; (ii) genome-quality-aware viral detection with CheckV; and (iii) machine learning–based classification with geNomad. Across these methods, 2,222 contigs were assigned to bacteriophage families by BLASTx/MEGAN, 602 were classified as viral by CheckV, and 760 were identified as viral by geNomad. The overlap among the three methods is shown in a Venn diagram (S7 Fig). To maximize precision, we retained only contigs supported by at least two approaches, yielding 767 high-confidence phage contigs for downstream analyses.

**Fig 6:**
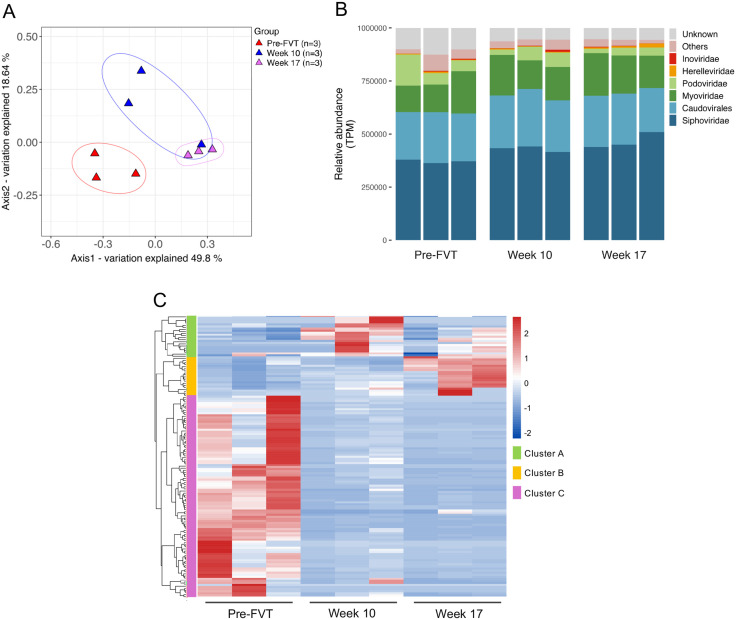
Temporal Dynamics and Differential Abundance of the Virome (767 high-confidence phage contigs) in the FVT group. (A) Beta diversity analysis of the virome using Bray-Curtis distances at different times pre- and post-FVT (n = 3, per time point). (B) Barplot of the relative abundance (normalized to counts per million, TPM) of viral families, categorized by sample for each time point. Unsupervised hierarchical clustering of the 136 differentially abundant contigs for week 10 and week 17 compared to pre-FVT (day 0). The corresponding heatmap displays the TPM relative abundances of each phage contig, as indicated by the color key. The corresponding heatmap displays Z-scores based on the relative abundance deviations of each phage contig across samples, as indicated by the color key. Contigs grouped in Cluster A represent those mostly increased at Week 10 compared with pre-FVT. Contigs in Cluster B represent those mostly increased at Week 17 compared with pre-FVT, while contigs in Cluster C correspond to those that decreased in abundance at both Week 10 and Week 17 relative to the pre-FVT timepoint.

The composition of the fecal viral community showed significant changes over time post-FVT. Specifically, the virome profiles at weeks 10 and 17 post-FVT were markedly different from the pre-FVT (Fig 6A). ANOSIM analysis confirmed a strong separation between the viral communities at these time points, with the differences being statistically significant (R = 0.654; p 0.006). This dynamic shift in the virome composition mirrored the alterations observed in the bacterial microbiota composition at the same corresponding post-FVT times (Fig 3B). However, no significant changes were detected in viral diversity (S8A in S8 Fig) and richness (S8B in S8 Fig) post-FVT, suggesting that although the virome composition shifted, its overall diversity and richness remained stable. The lack of significance may be attributable to the small n and consequent limited power; increasing the number of samples would enhance sensitivity to detect group differences.

Although geNomad applies the most recent ICTV taxonomy, in our dataset it provided assignments only up to the class level for 644 contigs and to the family level for 16 contigs, the remaining predictions were “virus” without a resolvable rank. To obtain a more interpretable family-level classification, we therefore relied on MEGAN applied to protein-level homology (BLASTx/DIAMOND) against curated viral protein databases. This approach assigned 702 contigs to viral families (Fig 6B). MEGAN’s conservative lowest common ancestor (LCA) algorithm is well-suited for fragmented contigs and provides transparent evidence for each taxonomic call, including alignment scores and support values. Of these 702 contigs, 513 and 609 were further validated as viral by CheckV and geNomad, respectively, reinforcing the robustness of their classification.. Most of these contigs belonged to the families Siphoviridae, Myoviridae, and the order Caudovirales (Fig 6B). Siphoviridae was the only family showing a significant change, with abundance increased at week 17 vs. pre-FVT (p < 0.05), consistent with a late-phase enrichment/stable engraftment following FVT.

We performed a contig-level differential-abundance test to examine virome changes at a finer resolution. Compared with pre-FVT, 29 contigs increased at week 10 (Fig 6C, Cluster A) and 23 at week 17 (Fig 6C, Cluster B), consistent with discrete, sustained phage expansions following FVT. Interestingly, 13 contigs were persistently increased at both weeks 10 and 17,suggesting a stable, FVT-responsive core virome over experimental time (Fig S9A). Additionally, 97 contigs were depleted at both weeks 10 and 17 compared to pre-FVT (Fig 6C, Cluster C), suggesting sustained post-FVT reductions.

To explore potential virome-bacteriome interactions, we correlated the abundances of the 13 viral contigs —overabundant at both time points (week 10 and week 17) — with the abundances of all bacterial taxa across samples. This analysis uncovered 4 phage contigs that were significantly correlated (Spearman correlation coefficient > 0.8; p < 0.05) with bacterial taxa, either positively or negatively (S9 Fig B). These correlations suggests potential phage-bacteria interactions contributing to the observed microbial community shifts following FVT.

To further understand the potential interactions between viruses and bacteria in shaping the observed metabolic phenotype, we performed host prediction analysis for our viral contigs. Bacterial host predictions were performed on 767 high-confidence phage contigs using IPHoP, and hosts could be predicted for 443 contigs (57.76%) with an average confidence level of 92.5% (S10 Fig). In total, 81 different bacterial genera were identified as potential hosts for these contigs. The most frequently predicted hosts were the genera Nanosyncoccus (9.9%), Bacteroides (9.4%), Oscillobacter (5%), Enterococcus (5%), and Acetatifactor (4.8%) (S10 Fig).

Notably, nine of the predicted bacterial genus hosts were also observed in our 16S rRNA dataset. These shared taxa accounted for predicted hosts of 21.67% of the viral contigs. Among them, members of the class Clostridia stood out as predicted hosts for multiple viral contigs. Notably, two of these contigs—scaffold_1599 (Myoviridae) and scaffold_856 (Caudovirales)—showed positive correlations with Clostridia abundance (Supplementary Fig S9 B), although this class was not differentially abundant in any group. These findings suggest the existence of specific phage–host interactions that may contribute to the shaping of the observed metabolic phenotype.

### 5. The overlap in microbiota may facilitate the colonization of human-derived phages in the recipient murine gut

**Fig 7:**
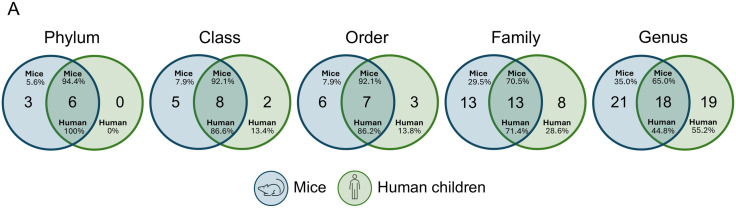
Overlap of bacterial taxa between human VLP donors and recipient mice. Venn diagrams (at the indicated taxonomic ranks) show shared vs. unique taxa. The numbers within each circle denote taxa unique to humans (blue) or mice (green); the intersection indicates shared taxa. The percentages reflect the cumulative relative abundance by the shared taxa within each cohort. Only taxa with relative abundance >0.01% were included.

Because phage engraftment requires compatible bacterial hosts, we assessed the overlap of the microbiota between the human VLPs donors and mice recipients. Notably, dominant taxa were widely shared between both microbiotas (Fig 7). For instance, at the genus level, the 18 genera common in both cohorts accounted for 65.0% of the murine community and 44.8% of the human community (Fig 7). This taxonomic concordance suggests that donor phages likely encountered permissive hosts in the mouse gut, consistent with the post-FVT shifts observed in the murine virome and microbiota.

### 6. Bacterial signal in the human derived VLP inoculum

We analyzed the bacterial signal in the human-derived VLP inoculum. To this end, we amplified the 16S rRNA V3–V4 region from total nucleic acids extracted from the VLP preparation. The PCR yielded a faint amplicon, indicating a very low bacterial-DNA fraction. After sequencing, the taxonomic profiling showed a dominance of Pseudomonas (76.2%), followed by Listeria seeligeri (6.8%), Bacillus spp. (6.7%), and Enterobacteriaceae (4.5%); all other taxa were <1%. However, because 16S profiling cannot distinguish DNA from viable cells vs. dead bacteria or extracellular DNA (and may include trace reagent contaminants), these signals should not be interpreted as evidence of live bacteria in the inoculum. In parallel, saline controls were carried out through the same extraction and PCR workflow. They produced no visible 16S band and negligible read counts (below our QC threshold). Finally, to test whether bacterial DNA detected in the VLP inoculum appeared in the mice recipients, we compared the inoculum profile with fecal 16S profiles from all mice. The only shared genus was Lactobacillus (0.5% in the inoculum), which was already present at baseline in both groups (mean 7%). It did not increase after inoculation (no within-group changes and no FVT–Control differences). Thus, any bacterial DNA associated with the inoculum did not engraft at levels detectable by our method. Together, these data suggest that any bacterial DNA associated with the VLP preparation was minimal and did not engraft at levels detectable by our methods.

## Discussion

We investigated the effects of the FVT from healthy human donors on mice with an HFD-induced obesity and MetS. Notably, FVT led to a significant improvement in glucose tolerance in the treated mice, despite continuous exposure to the HFD. Notably, the FVT and control groups exhibited similar body weights throughout the study, likely due to the prolonged HFD exposure—14 weeks pre- to and 17 weeks post-FVT, which may have exacerbated the metabolic dysfunction and limited the potential for weight modulation. While previous studies have shown that FVT can influence body composition, inducing lean or obese phenotypes in mice even under HFD conditions [11,13], several key methodological differences may explain our study’s lack of weight changes. Most notably, prior work used mice as donors and recipients, whereas our study uniquely utilized human-derived VLPs. Additionally, the duration of HFD exposure in our model (14 weeks pre-FVT) was substantially longer than in other studies (typically six weeks), which may have exacerbated disease severity and limited the capacity for weight modulation. Furthermore, the dosage and delivery of VLPs differed considerably; we administered a single oral dose of 4.4 × 10^8^ VLPs, while previous studies used doses approximately 100-fold higher and employed multiple inoculations [11,13]. These differences likely contributed to the divergent outcomes in body weight modulation. Despite these variations, our study and that of Rasmussen et al. [11] observed a delayed but significant improvement in glucose tolerance following FVT, by week 17 in our model and week 6 in theirs. This suggest that FVT in HFD-induced obesity and MetS mouse models may exert its metabolic benefits primarily through modulation of glucose homeostasis rather than weight reduction [31].

We evaluated whether FVT attenuates metabolic-syndrome traits under continuous high-fat feeding, modeling a real-world scenario in which the obesogenic diet is sustained and any benefits can be attributed solely to the virome. Previous studies have shown that switching from an HFD to a regular diet leads to a rapid reduction in weight gain and improvements in metabolic indices in mice [13]. Therefore, we maintained the HFD to isolate the effects of the virome. We recognize the importance of studying the virome’s impact in conjunction with a low-fat diet to understand the interactions between diet and FVT better, and we plan to include this in our future research.

Child donors (7–10 years) were intentionally not age-matched to recipient mice. Our goal was to test whether a well-defined human virome can remodel gut ecology and persist in the murine intestine irrespective of donor–host age equivalence; the observed bacterial and virome shifts support age-independent engraftment and broaden translational relevance. We acknowledge that donor and host age can influence colonization, and standardization strategies to mitigate such variability have been proposed [32].The mouse model used in our study develops obesity and MetS through an HFD, characterized by increased body weight, glucose intolerance, insulin resistance, elevated low-density lipoprotein (LDL) cholesterol, and hepatic lipid accumulation [19]. In addition to these metabolic alterations, HFD significantly disrupts the gut microbiota, reducing bacterial diversity and altering community composition [19]. Consistent with these observations, we found that mice in the FVT group exhibited significant shifts in gut microbiota composition as early as one week after receiving human-derived VLPs. In contrast, the microbiota in the control group remained stable (Fig 3).

Previous studies in gnotobiotic mice demonstrated that VLP inoculation [33] or the administration of specific lytic bacteriophages [34] can induce rapid changes in gut bacterial communities, with significant alterations observed within the first week post-inoculation. However, these studies typically monitored microbiota dynamics for only 2–3 weeks. In another experiment, inoculating VLPs from obese mice into normal-weight mice led to an obese-like microbiota phenotype in the ileum within four days post-FVT [35]. Importantly, our study shows that FVT-induced changes in the microbiota appeared at week 10 and were maintained for at least 17 weeks post-FVT (Fig 3C), providing observation of long-term microbiome modulation following a single human-to-mouse VLP transfer. This sustained effect highlights the potential of FVT to durably reshape host microbial communities, even in the context of ongoing HFD-induced metabolic stress.

Unlike bacterial-centered fecal microbiota transplantation (FMT), the FVT delivers replication-competent bacteriophages that can establish themselves in the gut. Once the initial infections occur, the phage populations can expand in situ, effectively self-amplifying the introduced inoculum [36]. In this proof-of-concept study, we administered a single dose of FVT to assess its efficacy and durability. The donor-derived phages successfully engrafted in the gut, maintaining substantial changes in the microbiota and virome for 17 weeks post-gavage. This data suggests that a single dose of FVT treatment can be effective, which aligns with murine studies demonstrating durable virome engraftment and phenotypic effects following a single administration of FVT [12]. However, multi-dose regimens may further enhance the benefits, as indicated by mouse-to-mouse FVT experiments [13,31]. We plan to investigate this potential advantage in our follow-up research.

While the gut microbiome—particularly its bacterial component—has been extensively studied for its role in host metabolism and obesity-related traits emerging evidence highlights the importance of the gut virome, particularly bacteriophages (phages), in shaping microbial dynamics and influencing metabolic outcomes. Phages change bacterial populations through either lytic or lysogenic cycles; in the lytic cycle, they destroy their hosts, while in the lysogenic cycle, they integrate into the bacterial genome as prophages. During infection, phage-encoded enzymes such as bacteriophage RNA polymerase that undergo major conformational changes that are essential for transcriptional regulation of the host takeover process [37].

In this study, we observed that *A. muciniphila* increased in the control group over the 17 weeks (Fig 3C), likely due to the prolonged high-fat diet (HFD). This finding is consistent with previous studies that have noted an increase in *A. muciniphila* abundance following prolonged HFD feeding [38]. In our study, mice were fed HFD for a total of 31 weeks, and a prolonged diet low in fiber, such as the one used in this study (6% fiber), could lead to the overabundance of *A. muciniphila*, which thrives by degrading mucins in the intestinal mucosal barrier [39]. This intestinal mucosal degradation has been linked to MetS, inflammation, and increased infection susceptibility [40–42]. Interestingly, in the FVT group, a contrary effect occurred with a significant decrease in *A. muciniphila* post-FVT (Fig 4A), suggesting that the VLPs in the transplant may counteract the effects of the HFD on this bacterium.

Also, we found a significant positive correlation between *A. muciniphila* and AUC glucose levels (Fig 4C), which aligns with prior studies that identified *A. muciniphila* as overabundant in patients with type 2 diabetes [43]. This suggests that the reduction of this bacterium in the FVT group may have contributed to the observed improvements in glucose tolerance. Also, an overabundance of *A. muciniphila* has been associated with other diseases, such as, ulcerative colitis [44], colorectal cancer [45], Parkinson’s [46], and multiple sclerosis [47].

Our findings were also supported by the observed changes in *Mucispirillum schaedleri*, which showed an initial increase in abundance in the FVT group at week 1 post-FVT (Fig 3C). *M. schaedleri* has been implicated in HFD-induced inflammation in mice [38,48], and its overabundance may be associated with prolonged exposure to HFD, as used in our study. However, by week 1, the abundance of *M. schaedleri* significantly decreased in the FVT group, which may have played a role in improving glucose tolerance. This reduction in both *A. muciniphila* and *M. schaedleri* could be indicative of a potential lytic effect of FVT on these bacteria or could reflect changes in the broader microbiota resulting from the transplantation of VLPs, which affected the population dynamics of phage-susceptible bacteria [34].

In contrast, Allobaculum showed a significant increase in abundance in the FVT group at week 17 (Fig 3C and 5A). This bacterium has been shown to improve metabolic dysfunction in obese mice and rats [49,50], possibly by producing short-chain fatty acids, which benefit metabolism [51]. Allobaculum also reduces pro-inflammatory markers such as IL-1β and TNF [50]. Moreover, Allobaculum has demonstrated high glucose utilization capacity [52], and in our study, its abundance was negatively correlated with the AUC of glucose levels (Fig 5C), indicating its potential role in improving glucose tolerance and supporting the positive metabolic effects observed in the FVT group.

We also observed an overabundance of Coprococcus in the FVT group at all time points post-FVT, with a significant increase at week 17 (Fig 3C). Studies have shown that an overabundance of Coprococcus can improve indicators in mental health [47] and has also been found to promote the desire to exercise in mice [53]. Furthermore, Coprococcus has been proposed as a potential probiotic for Inflammatory Bowel Disease (IBD) [54]. Based on these findings, the increase of Coprococcus may have contributed to alleviating some of the metabolic disturbances associated with MetS in the FVT group.

Interestingly, we observed a significant decrease in the abundance of Peptococcaceae at week 17 (Fig 3C). Previous studies have indicated that lower levels of this family are associated with higher HDL-cholesterol (HDL-c) [55]. In contrast, elevated levels of Peptococcaceae have been positively correlated with insulin resistance [56]. Given that insulin resistance is linked to poorer metabolic health and several cardiometabolic risk factors, including reduced HDL-c [57], it follows that lower levels of Peptococcaceae and higher HDL-c are indicative of better metabolic health [58]. Our findings suggest that the decrease in Peptococcaceae abundance could reflect a more favorable metabolic profile.

The human gut virome can vary greatly between individuals [6]. To ensure the diversity of the VLP inoculum used for FVT, we pooled VLPs from multiple healthy human donors, which successfully altered the virome of the recipient mice (Fig 6). Importantly, no bacteria were observed by epifluorescence microscopy (S1 Fig) and no cultivable bacteria were detected in the VLP inoculum (data not shown), confirming that the observed microbiota changes were primarily due to the VLPs and not residual fecal bacteria.

Regarding virome diversity, we observed that virome richness decreased in the FVT group at week 10 post-FVT but recovered by week 17 to baseline levels (S8B in S8 Fig), while virome diversity slightly increased at week 10 and remained stable until week 17 (S8A in S8 Fig). However, these changes were not statistically significant. At the viral family level, we identified that the most common families were Siphoviridae, Myoviridae, and Podoviridae (Fig 6B), which have been previously found in both mouse and human viromes. While Podoviridae was more abundant in other murine viromes [59], Herelleviridae and Inoviridae were detected at lower levels (Fig 6B), which have not been previously reported in virome studies [59]. Although we did not detect significant changes in the abundance of viral families over time, specific phages were differentially abundant at weeks 10 and 17 (Fig 6C), indicating dynamic phage-bacterial interactions post-FVT.

Previous studies have demonstrated the efficacy of FVT in reducing *C. difficile* infection in humans [15] and improving glucose metabolism in individuals with MetS [7]. In murine models, FVT has shown the capacity to reduce bacterial overgrowth [35], improve glucose tolerance and weight regulation in type 2 diabetes [11], and modulate host immunity and metabolic markers in metabolic dysfunction-associated fatty liver disease (MAFLD) [31]. Notably, these studies utilized either mouse-to-mouse or human-to-human VLP transfers. A recent study showed that human VLPs could alter gut microbiota composition in humanized gnotobiotic mice with IBD [16]. However, our study is the first to show that human-derived VLPs can significantly modulate the microbiota, the virome, and metabolic function in conventional mice with native microbiota. These findings support the hypothesis that cross-kingdom interactions between human viromes and murine bacteriomes can influence host metabolism. Our results also indicate a convergence between the donor and recipient microbial communities over time (Fig 7), probably facilitating the engraftment of human-derived phages in mice. Variability among donors and the risk of co-transferring pathogens, including eukaryotic viruses, have limited the clinical application of FVT. Recently, methods such as solvent/detergent inactivation of enveloped viruses and chemostat-based propagation to standardize donor material have maintained efficacy while enhancing safety in murine models of C. difficile infection [32]. These developments suggest a practical path toward broader clinical use of FVT.

Most virome workflows enhance DNA yield through whole-genome amplification (WGA), typically using multiple-displacement amplification (MDA). However, MDA is associated with several well-documented artifacts: it can generate chimeras, result in non-linear coverage, and crucially for gut studies, strongly over-amplify small circular single-stranded DNA (ssDNA) genomes [60–63]. These biases distort taxonomic profiles in sample-specific and non-reproducible ways, making quantitative abalysis of viromes challenging [63]. To preserve the natural abundance relationships, we adopted the ultra-low-input tagmentation (TAG) protocol proposed by Duhaime et al. (2012) [64]. This approach omits any pre-amplification and produces quantitatively reliable libraries for double-stranded DNA, even from picogram quantities. While we acknowledge that TAG may under-represent circular ssDNA templates [65,66] it is essential to note that non-enveloped, tailed double-stranded DNA (dsDNA) bacteriophages dominate the human gut virome, accounting for approximately 90–95% of viral reads [6,67,68]. Therefore, our protocol captured the vast majority of ecologically relevant phage diversity while avoiding the quantitative distortions associated with MDA.

Finally, while our study provides promising proof of concept, it also highlights several limitations. First, we administered whole viromes rather than isolated phage strains, precluding the identification of specific phages as causative agents responsible for the observed effects. Second, while our FVT protocol minimizes bacterial carryover, it may allow fecal components >100 kDa—including metabolites or extracellular vesicles—to co-transfer, contributing to microbiome modulation. Some residual solutes may remain after filtration and could exert transient, immediate post-gavage effects. However, the sustained virome and microbiota shifts observed over the weeks post-FVT were unlikely to be driven by short-lived metabolites present only at the time of the single gavage. Future work will include untargeted metabolomics of VLP preparations to quantify any residual small molecules. Furthermore, our study consists of two groups (FVT and control), and each mouse was observed for 17 weeks with repeated physiological, microbiota, and virome measurements. This longitudinal approach improves the precision of our findings compared to analyses performed at a single time point, and it helps mitigate the limitations posed by our relatively small sample size. Additionally, our DIO model showed homogeneous gut microbiota analysis with mice n = 6. Our primary endpoint and variance estimates from pilot/archival DIO data indicated that effects in this model are typically large (≥1 SD), for which n = 3–6 mice per group achieves 80% power at a = 0.05 [19]. Furthermore, our study was consistent with previous FVT research on mice, which typically involved 7–8 samples per group [11,12,32,33]. This design enables direct comparisons while adhering to ethical and resource constraints; taken together, these considerations make n = 6 per group scientifically defensible and ethically responsible for this proof-of-concept study. Our results open exciting opportunities for using human-derived viromes in standard murine models to investigate host-microbiome-virome interactions and develop novel therapeutic strategies for metabolic diseases.

Our findings should also be interpreted in light of key conceptual limitations of the proposed model and its relationship to current understanding of gut microbiome dynamics in metabolic health. First, the use of a DIO mouse model colonized with human-derived viromes provides an approximation of human MetS and does not fully recapitulate the inter-individual variability and comorbidities that characterize the human condition. Second, the metabolic impact of FVT in our model arises from interactions between human phages and a murine native bacteriome, in which host-range restrictions and species-specific metabolic functions may differ substantially from those in the human gut. As a result, the observed improvements in glucose tolerance—occurring in the absence of weight loss and under continuous HFD exposure—are influenced by the pre-existing bacterial community structure and its susceptibility to modulation by the transferred virome, highlighting the bacteriome as a key determinant of FVT responsiveness. Third, although our whole-virome approach more closely reflects the complexity of donor viromes, it precludes attribution of effects to specific phage taxa or functions, and does not rule out contributions from residual non-viral fecal components below the filtration cutoff. Thus, while our data support the concept that human-derived phage communities can durably reshape gut ecology and metabolic readouts in mice without dietary intervention, they should be viewed as proof-of-concept findings that require validation in complementary models, targeted phage designs, and ultimately human intervention studies before direct translational conclusions can be drawn.

## Supporting information

S1 Table ASVs abundances across microbiota samples. (XLSX)

S1 Fig Epifluorescence microscopy of virus-like particles (VLPs) stained with SYBR Green I. (A) *E. coli* (size control) to verify depletion of bacterial-sized cells in VLP concentrates. (B) Rhizobium phage ph01 (positive control) showing the expected virion signal. (C) Human fecal VLPs from donor H-193 (FVT inoculum). (D–F) Mouse fecal VLPs collected (D) pre-FVT, (E) week 10 post-FVT, and (F) week 17 post-FVT. All images were acquired with identical illumination and exposure settings.
(PDF)

S2 Fig Body weight and Food consumption. (A) Mean body weight (g) ± SEM at baseline (Pre-FVT) and at Weeks 1, 10 and 17 post-FVT treatment (n = 6 mice per timepoint). (B) Mean food consumption (g) per cage ± SEM at baseline (Pre-FVT) and at Weeks 1, 10 and 17 post-FVT treatment (2 cages with 6 mice each). No significant differences were detected by t-test.
(PDF)

S3 Fig Insulin-resistance test pre and post-FVT. (A) Mean area (±SEM) under the glucose curve (AUC, 0–120 min) at baseline (pre-FVT) and at weeks 10 and 17 post-FVT in the FVT and Control groups (n = 6 per group, per time point). (B-C) Mean blood glucose (±SEM) during intraperitoneal insulin administration at 0, 15, 30, 60, and 120 min for the FVT (B) and Control (C) groups at baseline (pre-FVT), and at weeks 10 and 17 post-FVT. No significant differences were detected by t-test.
(PDF)

S4 Fig Rarefaction curve of observed ASVs in the bacteriome. (A) Observed ASVs rarefaction curves generated at a minimum sequencing depth corresponding to 75% of the smallest sample (9,661 reads), averaged across 10,000 iterations.
(PDF)

S5 Fig Changes in bacteriome alpha-diversity pre and post-FVT. Comparisons of the (A) Shannon index and (B) Chao1 index of the bacterial microbiota at baseline (Pre-FVT) and at Day 1, Weeks 1, 10 and 17 after FVT treatment. Data are represented as median ± interquartile range, with outliers shown as black points. The Wilcoxon test detected significant differences at Week 1 between the FVT and control groups. No significant longitudinal differences were detected within groups.
(PDF)

S6 Fig Dynamics of bacterial abundances over time in FVT and control groups. Relative abundances of (A) bacterial families and (B) bacterial genera at baseline (Pre-FVT), and at Day 1, Week 1, Week 10, and Week 17 after FVT treatment.
(PDF)

S7 Fig Bioinformatic workflow for obtaining high-confidence phage contigs. (A) Pipeline overview: (i) assembly of viral reads and removal of contigs smaller than 2 kb; (ii) filtering of eukaryotic viruses to focus on bacteriophages, followed by quality control of contig coverage using the reads; (iii) validation of phage contigs using three complementary approaches: BLASTx against nr-viral proteins with LCA assignment via MEGAN, CheckV, and GeNomad.
(PDF)

S8 Fig Changes in virome alpha-diversity after FVT treatment. Comparisons of the (A) Shannon index and (B) Chao1 index of the viral communities at baseline (Pre-FVT) and after FVT treatment at w eeks 10 and 17. Data are represented as median ± interquartile range. Statistical comparisons were performed using the Wilcoxon test, and no significant differences were detected.
(PDF)

S9 Fig Correlations between phage contigs and bacteria. (A) Thirty-nine phage contigs that increased post-FVT at Weeks 10 and 17 relative to Pre-FVT were used for correlation analysis with bacterial taxa. (B) Of these contigs, four showed significant Spearman correlations with five different bacterial taxa. Predicted hosts for these phage contigs are indicated within the boxes.
(PDF)

S10 Fig Abundance of the top 20 most abundant genera predicted as phage hosts by IPHoP. (A) In total, IPHoP detected 81 different genera as potential bacterial hosts of 443 phage contigs. The top 20 genera represent 69.3% of the contigs with predicted hosts (307 contigs).
(PDF)
