# Supplementary material for: Human-derived fecal virome transplantation (FVT) reshapes the murine gut microbiota and virome, enhancing glucose regulation
Source: PLoS One. 2025 Dec 5;20(12):e0337760. doi: 10.1371/journal.pone.0337760 (PMC12680211; doi:10.1371/journal.pone.0337760)
Supplement: S1 Fig — (A) E. coli (size control) to verify depletion of bacterial-sized cells in VLP concentrates. (B) Rhizobium phage ph01 (positive control) showing the expected virion signal. (C) Human fecal VLPs from donor H-193 (FVT inoculum). (D–F) Mouse fecal VLPs collected (D) pre-FVT, (E) week 10 post-FVT, and (F) week 17 post-FVT. All images were acquired with identical illumination and exposure settings. (PDF) [file pone.0337760.s002.pdf]

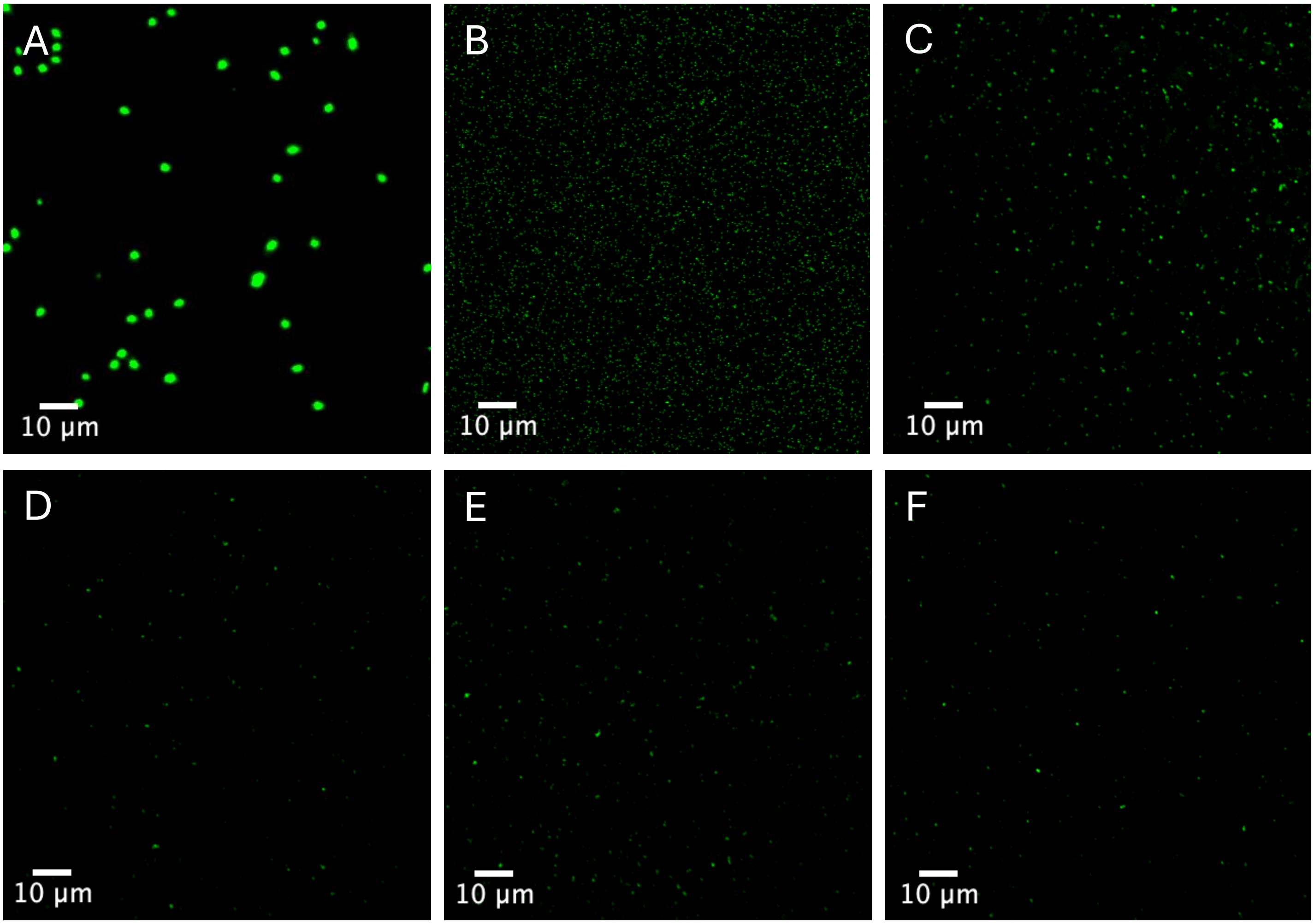

Figure. S1. Epifluorescence microscopy of virus-like particles (VLPs) stained with SYBR Green I. (A) Escherichia coli (size control) to verify depletion of bacterial-sized cells in VLP concentrates. (B) Rhizobium etli phage φ01 (positive control) showing the expected virion signal. (C) Human fecal VLPs from donor H-193 (FVT inoculum). (D–F) Mouse fecal VLPs collected (D) pre-FVT, (E) week 10 post-FVT, and (F) week 17 post-FVT. All images were acquired with identical illumination and exposure settings.
