# Supplementary material for: Human-derived fecal virome transplantation (FVT) reshapes the murine gut microbiota and virome, enhancing glucose regulation
Source: PLoS One. 2025 Dec 5;20(12):e0337760. doi: 10.1371/journal.pone.0337760 (PMC12680211; doi:10.1371/journal.pone.0337760)
Supplement: S2 Fig — (A) Mean body weight (g) ± SEM at baseline (Pre-FVT) and at Weeks 1, 10 and 17 post-FVT treatment (n = 6 mice per timepoint). (B) Mean food consumption (g) per cage ± SEM at baseline (Pre-FVT) and at Weeks 1, 10 and 17 post-FVT treatment (2 cages with 6 mice each). No significant differences were detected by t-test. (PDF) [file pone.0337760.s003.pdf]

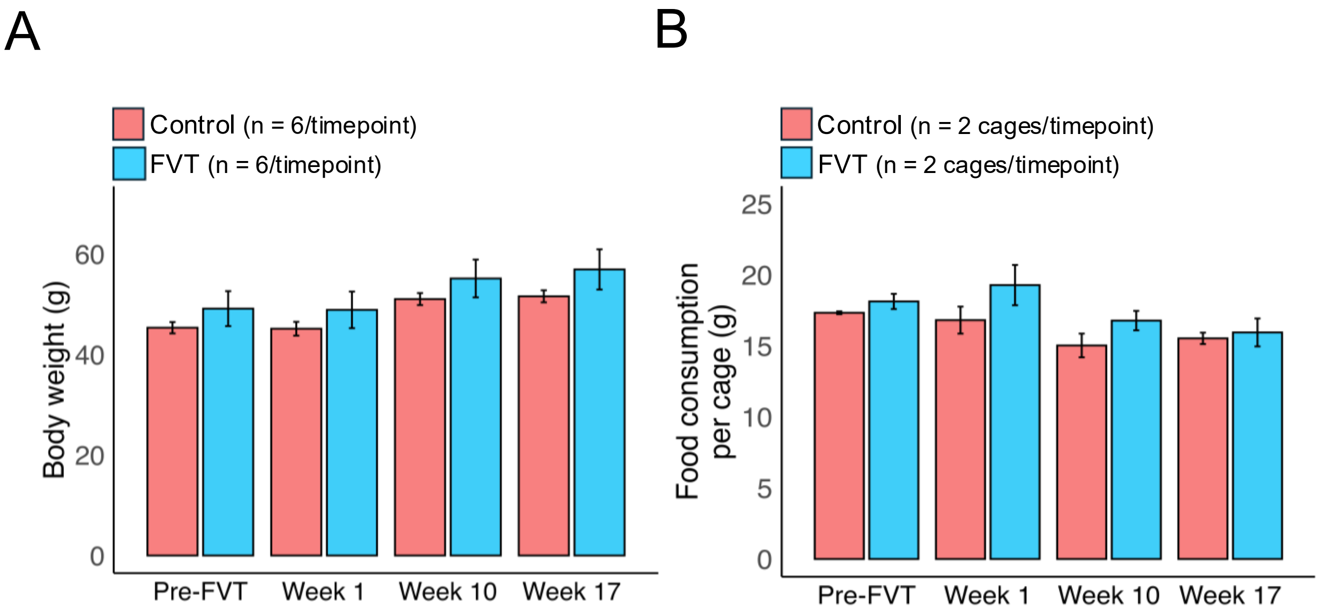

Figure S2. Body weight and Food consumption. (A) Mean body weight (g) ± SEM at baseline (Pre-FVT) and at Weeks 1, 10 and 17 post-FVT treatment (n = 6 mice per timepoint) . (B) Mean food consumption (g) per cage ± SEM at baseline (Pre-FVT) and at Weeks 1, 10 and 17 post-FVT treatment (2 cages with 6 mice each). No significant differences were detected by t-test.
