# Supplementary material for: Human-derived fecal virome transplantation (FVT) reshapes the murine gut microbiota and virome, enhancing glucose regulation
Source: PLoS One. 2025 Dec 5;20(12):e0337760. doi: 10.1371/journal.pone.0337760 (PMC12680211; doi:10.1371/journal.pone.0337760)
Supplement: S3 Fig — (A) Mean area (±SEM) under the glucose curve (AUC, 0–120 min) at baseline (pre-FVT) and at weeks 10 and 17 post-FVT in the FVT and Control groups (n = 6 per group, per time point). (B-C) Mean blood glucose (±SEM) during intraperitoneal insulin administration at 0, 15, 30, 60, and 120 min for the FVT (B) and Control (C) groups at baseline (pre-FVT), and at weeks 10 and 17 post-FVT. No significant differences were detected by t-test. (PDF) [file pone.0337760.s004.pdf]

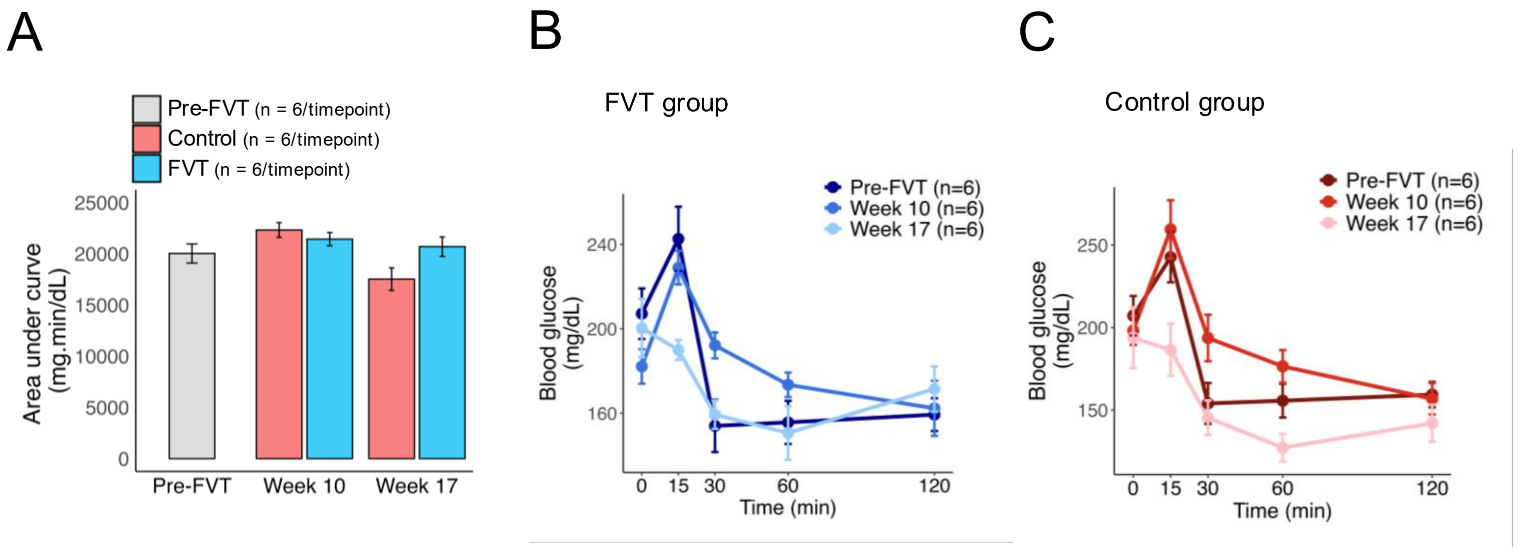

Figure S3. Insulin-resistance test pre and post-FVT. (A) Mean area (±SEM) under the glucose curve (AUC, 0-120 min) at baseline (pre-FVT) and at weeks 10 and 17 post-FVT in the FVT and Control groups (n = 6 per group, per time point). (B-C) Mean blood glucose (±SEM) during intraperitoneal insulin administration at 0, 15, 30, 60, and 120 min for the FVT (B) and Control (C) groups at baseline (pre-FVT), and at weeks 10 and 17 post-FVT. No significant differences were detected by t-test.
