# Supplementary material for: Human-derived fecal virome transplantation (FVT) reshapes the murine gut microbiota and virome, enhancing glucose regulation
Source: PLoS One. 2025 Dec 5;20(12):e0337760. doi: 10.1371/journal.pone.0337760 (PMC12680211; doi:10.1371/journal.pone.0337760)
Supplement: S4 Fig — (A) Observed ASVs rarefaction curves generated at a minimum sequencing depth corresponding to 75% of the smallest sample (9,661 reads), averaged across 10,000 iterations. (PDF) [file pone.0337760.s005.pdf]

A

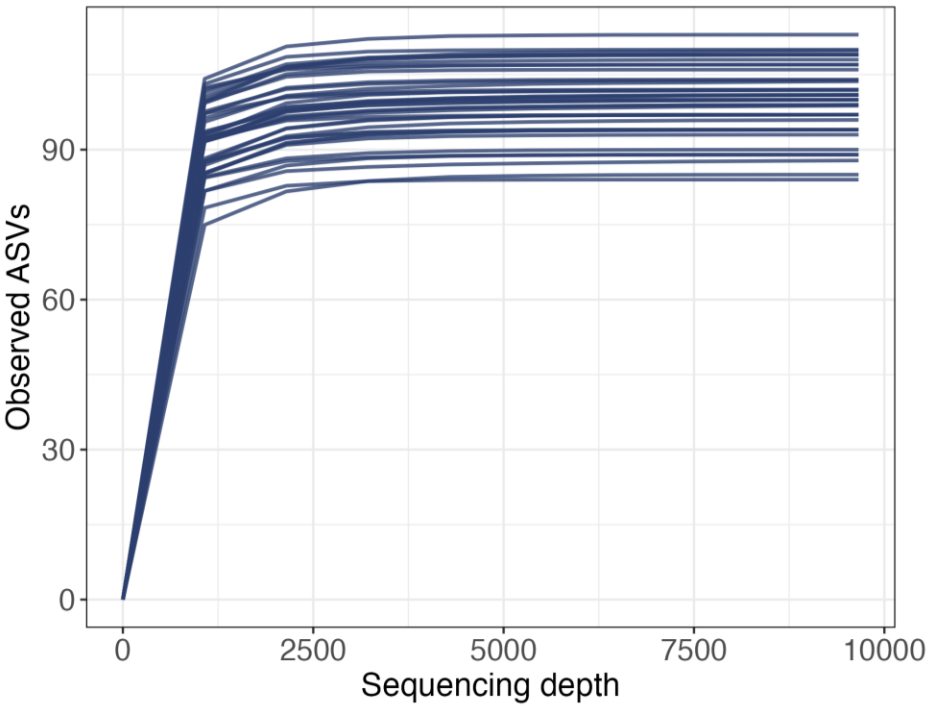


**Figure S4. Rarefaction curve of observed ASVs in the bacteriome.** (A) Observed ASVs rarefaction curves generated at a minimum sequencing depth corresponding to 75% of the smallest sample (9,661 reads), averaged across 10,000 iterations.
