# Supplementary material for: Human-derived fecal virome transplantation (FVT) reshapes the murine gut microbiota and virome, enhancing glucose regulation
Source: PLoS One. 2025 Dec 5;20(12):e0337760. doi: 10.1371/journal.pone.0337760 (PMC12680211; doi:10.1371/journal.pone.0337760)
Supplement: S5 Fig — Comparisons of the (A) Shannon index and (B) Chao1 index of the bacterial microbiota at baseline (Pre-FVT) and at Day 1, Weeks 1, 10 and 17 after FVT treatment. Data are represented as median ± interquartile range, with outliers shown as black points. The Wilcoxon test detected significant differences at Week 1 between the FVT and control groups. No significant longitudinal differences were detected within groups. (PDF) [file pone.0337760.s006.pdf]

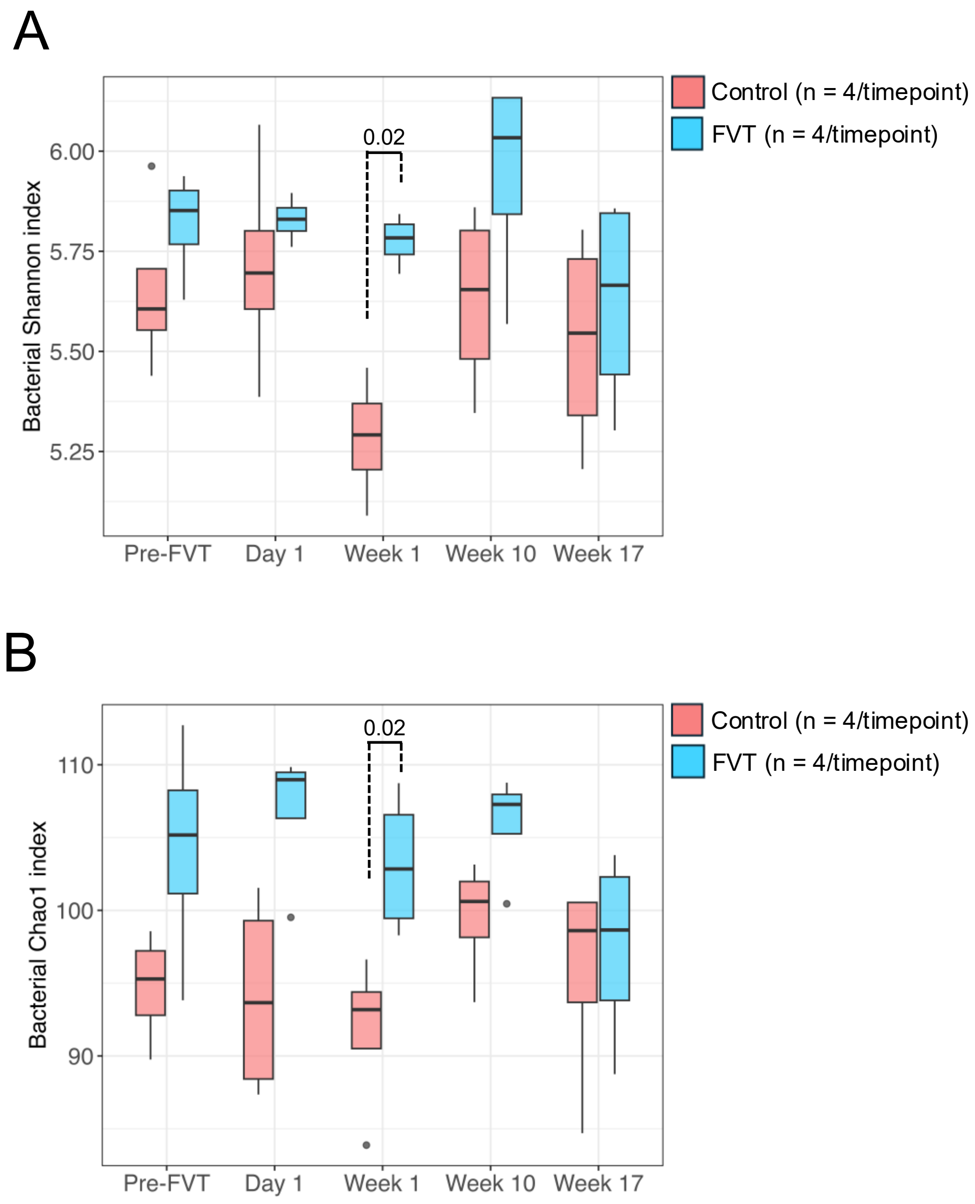

Figure S5. Changes in bacteriome alpha-diversity pre and post-FVT. Comparisons of the (A) Shannon index and (B) Chao1 index of the bacterial microbiota at baseline (Pre-FVT) and and at Day 1, Weeks 1, 10 and 17 after FVT treatment. Data are represented as median ± interquartile range, with outliers shown as black points. The Wilcoxon test detected significant differences at Week 1 between the FVT and control groups. No significant longitudinal differences were detected within groups.
