# Supplementary material for: Human-derived fecal virome transplantation (FVT) reshapes the murine gut microbiota and virome, enhancing glucose regulation
Source: PLoS One. 2025 Dec 5;20(12):e0337760. doi: 10.1371/journal.pone.0337760 (PMC12680211; doi:10.1371/journal.pone.0337760)
Supplement: S6 Fig — Relative abundances of (A) bacterial families and (B) bacterial genera at baseline (Pre-FVT), and at Day 1, Week 1, Week 10, and Week 17 after FVT treatment. (PDF) [file pone.0337760.s007.pdf]

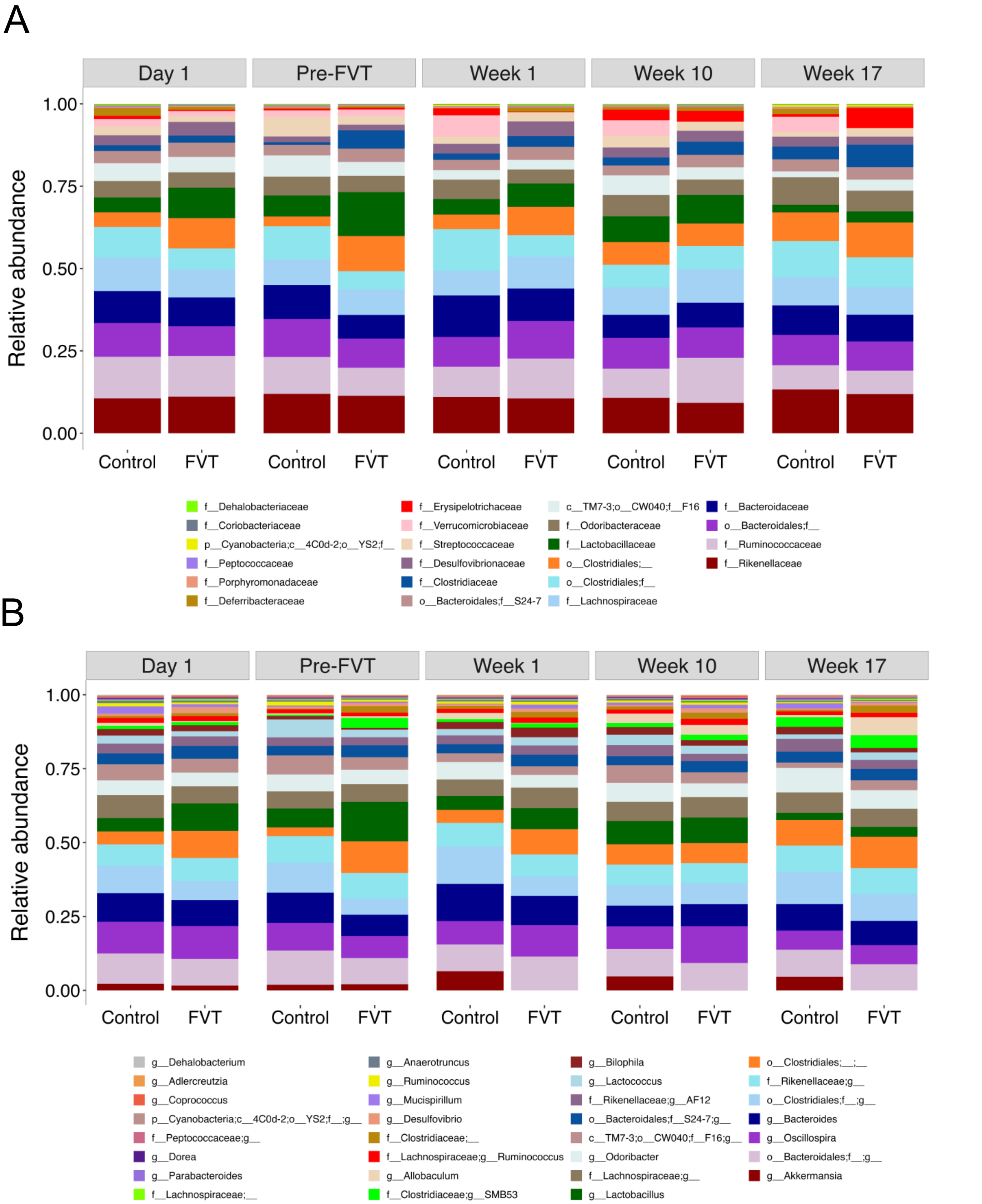

Figure S6. Dynamics of bacterial abundances over time in FVT and control groups. Relative abundances of (A) bacterial families and (B) bacterial genera at baseline (Pre-FVT), and at Day 1, Week 1, Week 10, and Week 17 after FVT treatment.
