# Supplementary material for: Human-derived fecal virome transplantation (FVT) reshapes the murine gut microbiota and virome, enhancing glucose regulation
Source: PLoS One. 2025 Dec 5;20(12):e0337760. doi: 10.1371/journal.pone.0337760 (PMC12680211; doi:10.1371/journal.pone.0337760)
Supplement: S7 Fig — (A) Pipeline overview: (i) assembly of viral reads and removal of contigs smaller than 2 kb; (ii) filtering of eukaryotic viruses to focus on bacteriophages, followed by quality control of contig coverage using the reads; (iii) validation of phage contigs using three complementary approaches: BLASTx against nr-viral proteins with LCA assignment via MEGAN, CheckV, and GeNomad. (PDF) [file pone.0337760.s008.pdf]

A

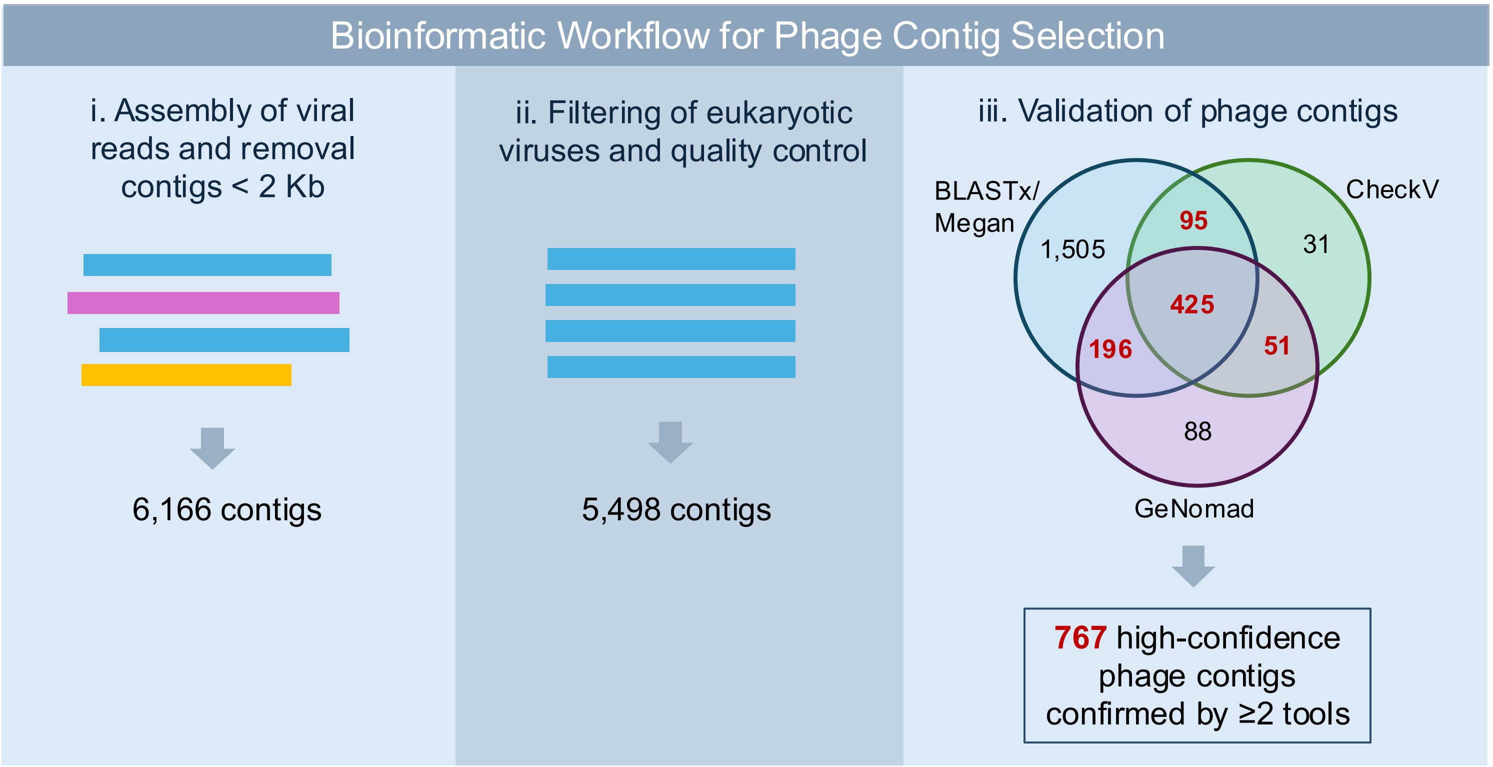

Figure S7. Bioinformatic workflow for obtaining high-confidence phage contigs. (A) Pipeline overview: (i) assembly of viral reads and removal of contigs smaller than 2 kb; (ii) filtering of eukaryotic viruses to focus on bacteriophages, followed by quality control of contig coverage using the reads; (iii) validation of phage contigs using three complementary approaches: BLASTx against nr-viral proteins with LCA assignment via MEGAN, CheckV, and GeNomad.
