# Supplementary material for: Human-derived fecal virome transplantation (FVT) reshapes the murine gut microbiota and virome, enhancing glucose regulation
Source: PLoS One. 2025 Dec 5;20(12):e0337760. doi: 10.1371/journal.pone.0337760 (PMC12680211; doi:10.1371/journal.pone.0337760)
Supplement: S8 Fig — Comparisons of the (A) Shannon index and (B) Chao1 index of the viral communities at baseline (Pre-FVT) and after FVT treatment at w eeks 10 and 17. Data are represented as median ± interquartile range. Statistical comparisons were performed using the Wilcoxon test, and no significant differences were detected. (PDF) [file pone.0337760.s009.pdf]

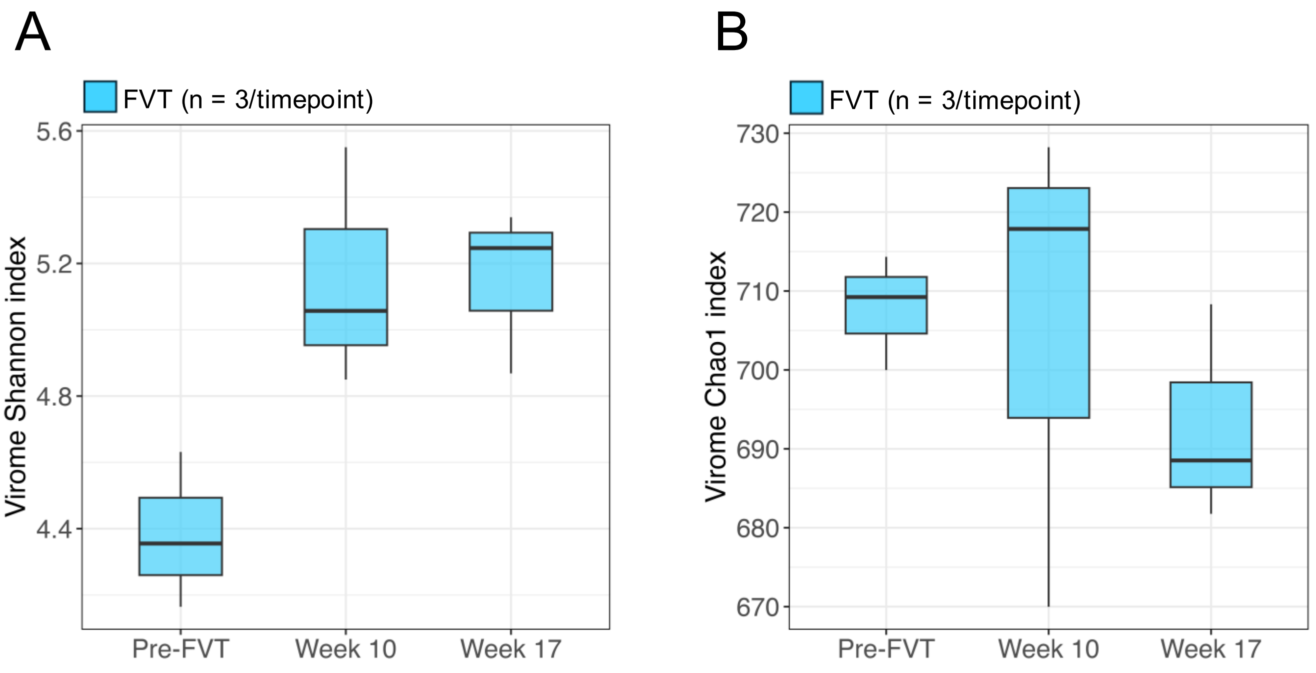

Figure S8. Changes in virome alpha-diversity after FVT treatment. Comparisons of the (A) Shannon index and (B) Chao1 index of the viral communities at baseline (Pre-FVT) and after FVT treatment at w eeks 10 and 17. Data are represented as median ± interquartile range. Statistical comparisons were performed using the Wilcoxon test, and no significant differences were detected
