# Supplementary material for: Human-derived fecal virome transplantation (FVT) reshapes the murine gut microbiota and virome, enhancing glucose regulation
Source: PLoS One. 2025 Dec 5;20(12):e0337760. doi: 10.1371/journal.pone.0337760 (PMC12680211; doi:10.1371/journal.pone.0337760)
Supplement: S9 Fig — (A) Thirty-nine phage contigs that increased post-FVT at Weeks 10 and 17 relative to Pre-FVT were used for correlation analysis with bacterial taxa. (B) Of these contigs, four showed significant Spearman correlations with five different bacterial taxa. Predicted hosts for these phage contigs are indicated within the boxes. (PDF) [file pone.0337760.s010.pdf]

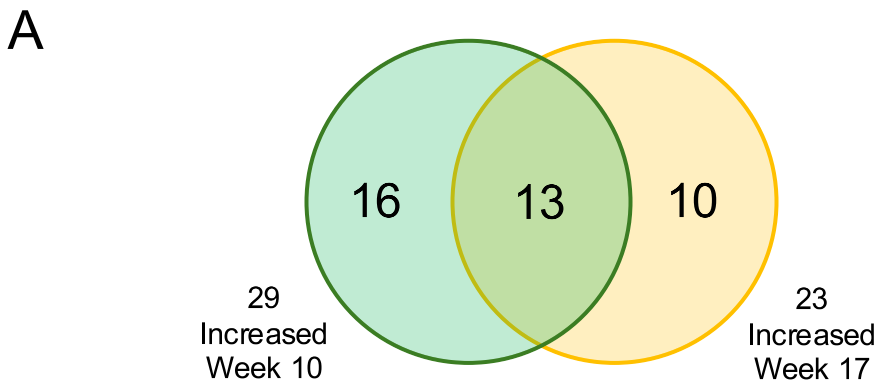

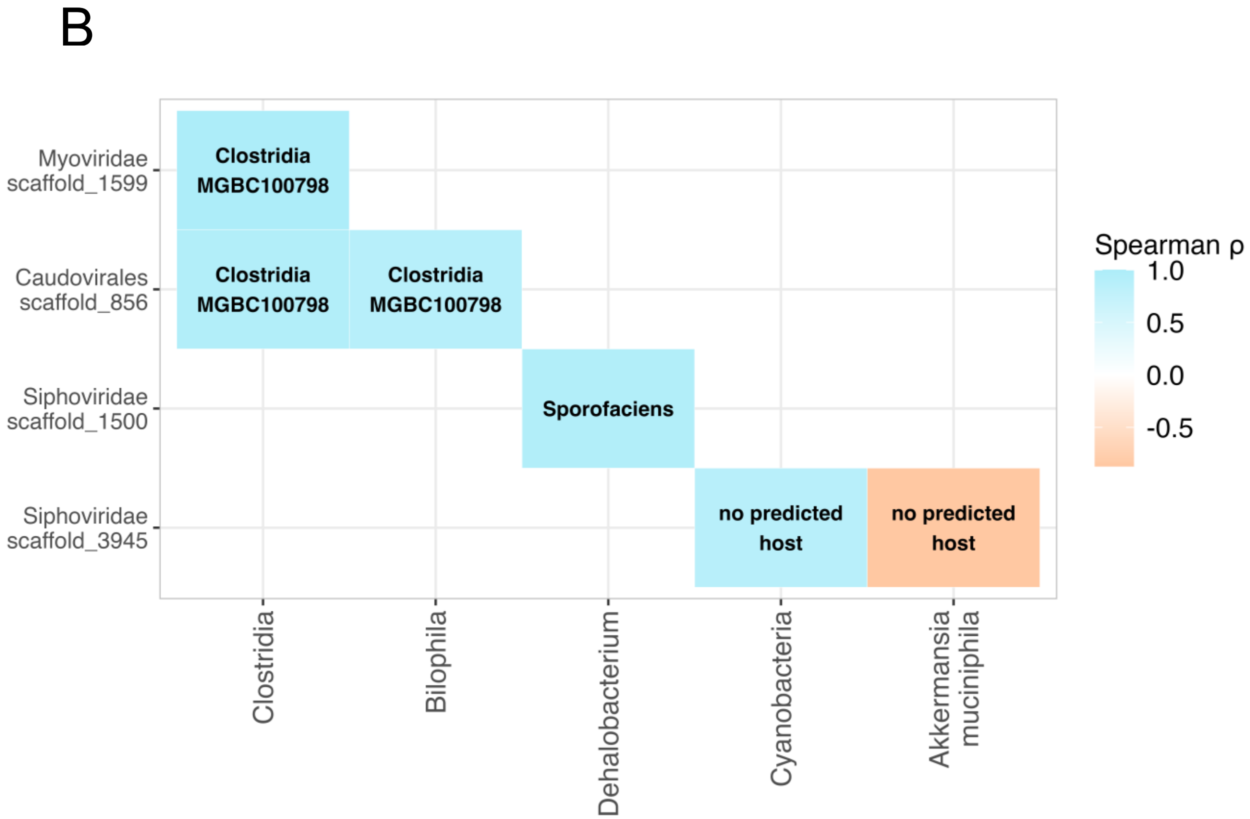

Figure S9. Correlations between phage contigs and bacteria. (A) Thirty-nine phage contigs that increased post-FVT at Weeks 10 and 17 relative to Pre-FVT were used for correlation analysis with bacterial taxa. (B) Of these contigs, four showed significant Spearman correlations with five different bacterial taxa. Predicted hosts for these phage contigs are indicated within the boxes.
