# Supplementary material for: Human-derived fecal virome transplantation (FVT) reshapes the murine gut microbiota and virome, enhancing glucose regulation
Source: PLoS One. 2025 Dec 5;20(12):e0337760. doi: 10.1371/journal.pone.0337760 (PMC12680211; doi:10.1371/journal.pone.0337760)
Supplement: S10 Fig — The top 20 genera represent 69.3% of the contigs with predicted hosts (307 contigs). (PDF) [file pone.0337760.s011.pdf]

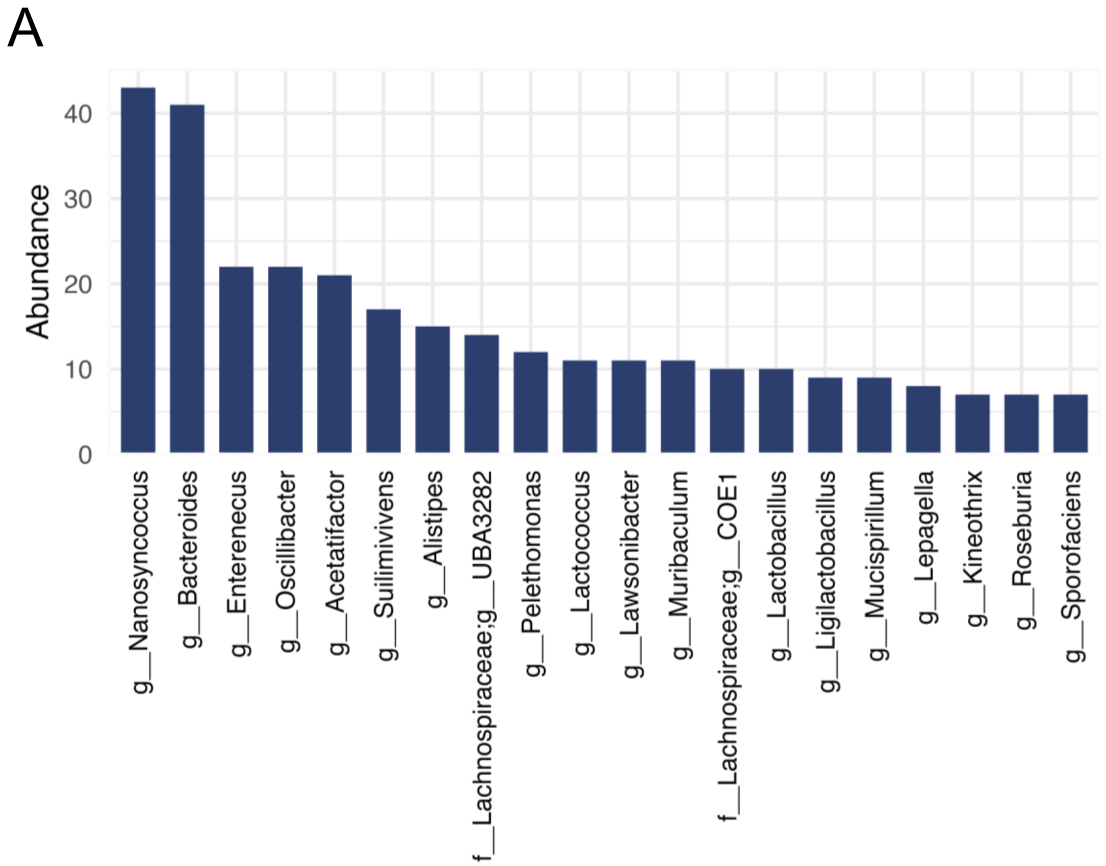

Figure S10. Abundance of the top 20 most abundant genera predicted as phage hosts by IPHoP. (A) In total, IPHoP detected 81 different genera as potential bacterial hosts of 443 phage contigs. The top 20 genera represent 69.3 % of the contigs with predicted hosts (307 contigs).
